# Test–Retest Reliability of the Effects of Continuous Theta-Burst Stimulation

**DOI:** 10.3389/fnins.2019.00447

**Published:** 2019-05-17

**Authors:** Ali Jannati, Peter J. Fried, Gabrielle Block, Lindsay M. Oberman, Alexander Rotenberg, Alvaro Pascual-Leone

**Affiliations:** ^1^Berenson-Allen Center for Noninvasive Brain Stimulation and Division of Cognitive Neurology, Department of Neurology, Beth Israel Deaconess Medical Center, Harvard Medical School, Boston, MA, United States; ^2^Neuroplasticity and Autism Spectrum Disorder Program, Department of Psychiatry and Human Behavior, E.P. Bradley Hospital, Warren Alpert Medical School, Brown University, East Providence, RI, United States; ^3^Neuromodulation Program and Division of Epilepsy and Clinical Neurophysiology, Department of Neurology, Boston Children’s Hospital, Harvard Medical School, Boston, MA, United States; ^4^Institut Guttman de Neurorehabilitació, Universitat Autónoma de Barcelona, Barcelona, Spain

**Keywords:** transcranial magnetic stimulation, continuous theta-burst stimulation, plasticity, reliability, *BDNF*, *APOE*

## Abstract

**Objectives:**

The utility of continuous theta-burst stimulation (cTBS) as index of cortical plasticity is limited by inadequate characterization of its test–retest reliability. We thus evaluated the reliability of cTBS aftereffects, and explored the roles of age and common single-nucleotide polymorphisms in the brain-derived neurotrophic factor (*BDNF*) and apolipoprotein E (*APOE*) genes.

**Methods:**

Twenty-eight healthy adults (age range 21–65) underwent two identical cTBS sessions (median interval = 9.5 days) targeting the motor cortex. Intraclass correlation coefficients (ICCs) of the log-transformed, baseline-corrected amplitude of motor evoked potentials (ΔMEP) at 5–60 min post-cTBS (T5–T60) were calculated. Adjusted effect sizes for cTBS aftereffects were then calculated by taking into account the reliability of each cTBS measure.

**Results:**

ΔMEP at T50 was the most-reliable cTBS measure in the whole sample (ICC = 0.53). Area under-the-curve (AUC) of ΔMEPs was most reliable when calculated over the full 60 min post-cTBS (ICC = 0.40). cTBS measures were substantially more reliable in younger participants (< 35 years) and in those with *BDNF* Val66Val and *APOE* ε4– genotypes.

**Conclusion:**

cTBS aftereffects are most reliable when assessed 50 min post-cTBS, or when cumulative ΔMEP measures are calculated over 30–60 min post-cTBS. Reliability of cTBS aftereffects is influenced by age, and *BDNF* and *APOE* polymorphisms. Reliability coefficients are used to adjust effect-size calculations for interpretation and planning of cTBS studies.

## Introduction

Transcranial magnetic stimulation (TMS) is a method for focal non-invasive stimulation of the brain through electromagnetic induction (Barker et al., 1985). Application of TMS within the recommended guidelines (Rossi et al., 2009; Rossini et al., 2015) is a safe means of triggering or modulating neural activity in a given brain region or network (Pascual-Leone et al., 2011; Fox et al., 2012; Valero-Cabré et al., 2017). A form of repetitive TMS (rTMS) known as continuous theta-burst stimulation (cTBS) consists of 50 Hz bursts of three TMS pulses repeated at 5 Hz for a total of 600 pulses over 40 s (Huang et al., 2005). The average amplitude of motor evoked potentials (MEPs) induced by single TMS pulses is reduced by approximately 25% for up to 50 min following cTBS of the primary motor cortex (M1) (Wischnewski and Schutter, 2015). This neuromodulatory effect is thought to involve mechanisms similar to long-term depression (LTD) (Pascual-Leone et al., 1994; Huang et al., 2005; Hallett, 2007). Therefore, the pattern of cTBS-induced changes in MEPs provides a neurophysiologic index of the mechanism of cortical plasticity (Pascual-Leone et al., 2005, 2011; Oberman et al., 2010, 2012, 2014, 2016; Suppa et al., 2016).

The neuromodulatory effect of cTBS applied to M1 or other brain regions has been investigated for its potential as a neurophysiological biomarker and a therapeutic intervention in several neurological and psychiatric disorders (Koch et al., 2009, 2012; Eberle et al., 2010; McClintock et al., 2011; Cazzoli et al., 2012; Oberman et al., 2012, 2014; Di Lazzaro et al., 2013, 2016; Mori et al., 2013; Cantone et al., 2014; Chuang et al., 2014; Forogh et al., 2014; Li et al., 2014; Suppa et al., 2014; Carrette et al., 2016). Despite its growing popularity, however, cTBS responses show large inter-individual (Hamada et al., 2013; Goldsworthy et al., 2014; López-Alonso et al., 2014; Vallence et al., 2015; Guerra et al., 2017; Heidegger et al., 2017; Hordacre et al., 2017; Jannati et al., 2017) and intra-individual variability (Vernet et al., 2014; Vallence et al., 2015) that can limit the utility of cTBS for assessing brain plasticity in clinical populations.

Only two published studies have assessed the reproducibility of cTBS aftereffects (Vernet et al., 2014; Vallence et al., 2015). The first (Vernet et al., 2014) used a relatively small sample size (*n* = 10) and did not report reliability coefficients of cTBS aftereffects, which can be compared with the reliability coefficients of other TMS measures (Carroll et al., 2001; Kimiskidis et al., 2004; Christie et al., 2007; Livingston and Ingersoll, 2008; Bastani and Jaberzadeh, 2012; Ngomo et al., 2012; Hinder et al., 2014; Liu and Au-Yeung, 2014; Sankarasubramanian et al., 2015; Schambra et al., 2015; Hermsen et al., 2016; Fried et al., 2017). The second study (Vallence et al., 2015) used an input–output curve approach that allowed assessment of cTBS aftereffects elicited over a range of stimulation intensities, but at the cost of fewer time-points. Specifically, assessments were only performed at 0, 15, and 30 min post-cTBS. This excluded the earliest time points, i.e., 5 and 10 min post-cTBS, which typically exhibit the maximal cTBS effects (Wischnewski and Schutter, 2015) and later time-points, i.e., 40–60 min post-cTBS, which capture the longer-lasting TBS effects and have been found to be useful in differentiating clinical populations such as individuals with Alzheimer’s disease (Freitas et al., 2011), autism spectrum disorder (Oberman et al., 2012), diabetes (Fried et al., 2016), and schizophrenia (McClintock et al., 2011) from healthy individuals. For comparison, at least three studies have assessed the reliability of intermittent theta-burst stimulation (iTBS) aftereffects (Hinder et al., 2014; Fried et al., 2017; Schilberg et al., 2017).

Full characterization of the test–retest reliability of cTBS aftereffects is essential to properly interpret results and plan for future studies. We thus aimed to address this need by systematically assessing the test–retest reliability of cTBS aftereffects in 5- or 10-min intervals for 60 min post-cTBS in a sizeable sample of healthy adults. We also calculated adjusted effect sizes for cTBS aftereffects by taking into account the reliability (or lack thereof) of each cTBS measure (Friedman, 1968; Wright, 2014; Fried et al., 2017). In addition, we explored the effects of age group on the reproducibility of cTBS aftereffects, as well as of single-nucleotide polymorphisms (SNPs) in brain-derived neurotrophic factor (*BDNF*) and apolipoprotein E (*APOE*) genes, which have been found to influence neuroplasticity (White et al., 2001; Cheeran et al., 2008; Nichol et al., 2009; Antal et al., 2010; Peña-Gomez et al., 2012; Lee et al., 2013; Chang et al., 2014; Di Lazzaro et al., 2015; Jannati et al., 2017). Our results can improve the utility of cTBS as a neurophysiologic index of cortical plasticity in neurological and psychiatric disorders, help elucidate the sources of intra-individual variability in cTBS responses, and ensure adequate sample size and power in future cTBS studies in clinical populations.

## Materials and Methods

### Participants

Twenty-eight healthy adults (25 males, age range: 21–65) participated in the study, which was approved by the local Institutional Review Board in accordance with the Declaration of Helsinki. All participants provided written informed consent prior to enrollment and received monetary compensation upon completion. None of the participants had any TMS contraindication (Rossi et al., 2009), and all had normal physical and neurological examinations. Individual and group-level demographics are presented in Tables 1 and 2, respectively.

**Table 1:** Participants’ demographics, neuropsychological measures, single-nucleotide polymorphisms, and inter-visit measures.

ID	Age range (years)	Race	Ethnicity	Education (year)	Handedness	*BDNF*	*APOE*	MMSE	IQ	Verbal KN	Non-verbal FR	Inter-visit interval (days)	Visit-A start time	Visit-B start time	|Δ_B-A_| start time (min)
1	20–25	White	Non-Hispanic	16	R	Val/Met	ε3/ε3	30	130	17	13	10	10:20	11:18	58
2	26–30	Other	Non-Hispanic	19	R	Val/Val	ε3/ε4	30	121	17	10	8	13:50	13:50	0
3	20–25	Multiracial	Hispanic	16	R	Val/Val	ε2/ε4	30	106	11	11	33	14:50	14:52	2
4	20–25	Multiracial	Non-Hispanic	16	R	–	–	30	112	11	13	7	9:55	9:50	5
5	56–60	White	Non-Hispanic	16	R	Val/Met	ε3/ε3	30	109	13	10	7	9:36	9:25	11
6	56–60	Black	Non-Hispanic	14	R	–	–	30	97	9	10	28	10:50	11:43	53
7	46–50	White	Non-Hispanic	20+	R	Val/Val	ε3/ε3	30	133	17	14	9	14:38	13:57	41
8	20–25	Asian	Non-Hispanic	16	R	–	–	30	94	8	10	7	9:27	9:39	12
9	50–55	White	Non-Hispanic	16	R	Val/Val	ε3/ε4	30	106	13	9	9	14:00	14:01	1
10	50–55	White	Non-Hispanic	16	R	Val/Val	ε3/ε3	30	115	14	11	19	12:56	14:39	103
11	60–65	White	Non-Hispanic	16	R	Val/Met	ε3/ε4	30	118	12	14	7	11:25	11:22	3
12	20–25	White	Hispanic	15	R	Val/Val	ε3/ε4	30	109	14	9	10	9:42	9:52	10
13	20–25	White	Hispanic	17	R	Val/Val	ε2/ε3	30	112	10	14	7	9:47	9:39	8
14	30–35	White	Non-Hispanic	19	R	Val/Val	ε3/ε3	29	100	9	11	8	14:33	10:19	254
15	20–25	White	Non-Hispanic	17	R	–	–	30	118	13	13	8	9:46	9:28	18
16	45–50	White	Non-Hispanic	16	R	–	–	30	94	9	9	28	14:04	13:36	28
17	26–30	White	Hispanic	–	R	Val/Val	ε3/ε3	30	115	13	12	12	12:04	13:10	66
18	26–30	White	Hispanic	–	R	Val/Val	ε3/ε3	29	100	10	10	7	10:52	10:50	2
19	50–55	Black	Non-Hispanic	–	R	–	–	30	97	10	9	11	9:12	12:07	175
20	20–25	White	Non-Hispanic	17	R	Val/Val	ε2/ε3	30	127	16	13	7	14:14	14:15	1
21	20–25	Multiracial	Hispanic	17	R	Val/Met	ε2/ε3	28	115	12	13	7	9:50	10:14	24
22	45–50	Black	Non-Hispanic	18	R	Val/Val	ε3/ε4	29	118	16	10	14	11:29	14:09	160
23	20–25	Asian	Non-Hispanic	17	R	Val/Met	ε3/ε4	30	103	9	12	17	9:41	9:40	1
24	45–50	Black	Non-Hispanic	18	R	Val/Met	ε3/ε3	30	88	9	7	19	10:43	10:45	2
25	30–35	Asian	Non-Hispanic	20+	R	Val/Met	ε3/ε4	30	103	8	13	14	9:53	9:41	12
26	60–65	Asian	Non-Hispanic	20+	R	Val/Val	ε3/ε4	30	124	15	13	7	11:02	10:04	58
27	45–50	White	Non-Hispanic	13	R	Val/Val	ε3/ε4	30	88	9	7	19	11:47	13:02	75
28	30–35	White	Non-Hispanic	20+	L	Val/Met	ε3/ε4	29	97	10	9	20	13:55	13:48	7


*|Δ_B-A_|, absolute inter-visit difference; APOE, apolipoprotein E; BDNF, brain derived neurotrophic factor; FR, fluid reasoning; IQ, intelligence quotient; KN, knowledge; Met, methionine; MMSE, Mini-Mental State Examination; Val, valine. Racial and ethnic categories were defined according to the National Institutes of Health policy and guidelines on the inclusion of minorities as subjects in clinical research (NIH Office of Extramural Research, 2001).*

**Table 2:** Participants’ demographics, single-nucleotide polymorphisms, neuropsychological results, and neurophysiological measures for the total sample and for the age and genetic subgroups.

	All (*N* = 28)	Age < 35 (*n* = 16)	Age ≥ 45 (*n* = 12)	*p*	*BDNF* Met- (*n* = 14)^†^	*BDNF* Met+ (*n* = 8)^†^	*p*	*APOE* ε4- (*n* = 12)^†^	*APOE* ε4+ (*n* = 10)^†^	*p*
Age (year, mean ± SD)	36.8 ± 14.5	25.3 ± 4.3	52.1 ± 6.5	N/ A	36.6 ± 14.4	37.8 ± 15.4	0.86	33.8 ± 12.6	40.8 ± 16.1	0.27
Sex (M : F)	25 : 3	15 : 1	10 : 2	0.56	13 : 1	8 : 0	1.00	11 : 1	10 : 0	1
Race (White : non-White)	16 : 12	9 : 7	7 : 5	1.00	10 : 4	4 : 4	0.39	9 : 3	5 : 5	0.38
Ethnicity (Hispanic : non-Hispanic)	6 : 22	6 : 10	0 : 12	**0.02**	9 : 5	7 : 1	0.35	7 : 5	9 : 1	0.16
Education (year, mean ± SD)^∗^	17.0 ± 2.3	17.4 ± 1.9	16.4 ± 2.8	–	17.3 ± 2.4	17.8 ± 2.1	0.69	17.3 ± 1.6	17.7 ± 2.8	–
*BDNF* (Met- : Met+)^†^	14 : 8	8 : 5	6 : 3	–	14 : 0	0 : 8	N/A	8 : 4	6 : 4	1.00
*APOE* (ε4- : ε4+)^†^	12 : 10	8 : 5	4 : 5	–	8 : 6	4 : 4	1.00	12 : 0	0 : 10	N/ A
Handedness (Right: Left )	27 : 1	15 : 1	12 : 0	1.00	14 : 0	7 : 1	0.36	12 : 0	9 : 1	0.46
MMSE score (mean ± SD)	29.8 ± 0.5	29.7 ± 0.6	29.9 ± 0.3	0.24	29.8 ± 0.4	29.6 ± 0.7	0.52	29.7 ± 0.7	29.8 ± 0.4	0.58
IQ (mean ± SD)	108.9 ± 12.3	110.1 ± 10.6	107.2 ± 14.7	0.55	112.4 ± 12.0	107.9 ± 13.1	0.42	112.5 ± 13.2	108.7 ± 11.5	0.49
Verbal KN score	11.9 ± 2.9	11.8 ± 3.0	12.2 ± 2.9	0.71	13.1 ± 2.9	11.3 ± 2.9	0.16	12.6 ± 2.9	12.3 ± 3.2	0.83
Non-verbal FR score	11.0 ± 2.0	11.6 ± 1.6	10.3 ± 2.4	0.08	11.0 ± 2.0	11.4 ± 2.4	0.70	11.6 ± 2.0	10.6 ± 2.3	0.30
RMT (% MSO, mean ± SD)										
Visit A	35.3 ± 7.6	35.4 ± 8.7	35.3 ± 6.4	0.97	32.4 ± 5.5	38.1 ± 9.7	0.09	31.2 ± 6.0	37.9 ± 8.2	0.054
Visit B	35.9 ± 7.7	35.9 ± 8.2	35.9 ± 7.4	0.99	33.6 ± 5.8	37.4 ± 10.1	0.27	32.4 ± 6.4	38.0 ± 8.3	0.09
AMT (% MSO, mean ± SD)										
Visit A	25.9 ± 5.2	26.6 ± 5.9	25.0 ± 4.0	0.44	24.3 ± 3.7	27.3 ± 6.2	0.18	24.6 ± 4.8	26.3 ± 5.1	0.42
Visit B	25.7 ± 4.6	25.9 ± 5.0	25.4 ± 4.3	0.78	24.3 ± 3.2	26.8 ± 5.9	0.21	24.3 ± 4.2	26.3 ± 4.6	0.29
Baseline MEP amplitude (mV, mean ± SD)										
Visit A	1.3 ± 1.5	1.1 ± 1.1	1.5 ± 1.9	0.42	1.2 ± 1.2	0.8 ± 0.5	0.45	1.1 ± 1.3	1.0 ± 0.5	0.75
Visit B	1.1 ± 1.0	1.2 ± 1.0	1.1 ± 1.0	0.82	1.4 ± 1.0	0.7 ± 0.4	0.08	1.2 ± 1.1	0.9 ± 0.5	0.43
Intervisit interval (days) (mean ± SD)	12.8 ± 7.4	11.4 ± 7.0	14.8 ± 7.8	0.24	12.1 ± 7.3	12.6 ± 5.6	0.86	12.1 ± 7.9	12.5 ± 5.0	0.89
|Δ_B-A_| Start Time (min, mean ± SD)	42.5 ± 62.2	30.0 ± 62.9	59.2 ± 59.7	0.23	55.8 ± 74.6	14.8 ± 19.0	0.15	47.7 ± 72.6	32.7 ± 51.9	0.59


*Comparisons of proportions were conducted with Fisher’s exact test. Education and single-nucleotide polymorphisms were not statistically compared between the subgroups because the data were not available for the total sample. The *p*-values were not adjusted for multiple comparisons. The *p*-value < 0.05 is highlighted in bold font. |Δ_*B-A*_|, absolute inter-visit difference; AMT, active motor threshold; *APOE*, apolipoprotein E; *APOE* ε4+, ε2/ε4 or ε3/ε4 genotype; *APOE* ε4–, ε2/ε3 or ε3/ε3; *BDNF*, brain-derived neurotrophic factor; *BDNF* Met–, Val66Val; *BDNF* Met+, Val66 Met; FR, fluid reasoning; IQ, intelligence quotient; KN, knowledge; MEP, motor evoked potential; Met, metionine; MMSE, Mini-Mental State Examination; MSO, maximum stimulator output; RMT, resting motor threshold; SD, standard deviation; Val, valine. ^∗^Education data were available for 26 participants. ^†^*BDNF* and *APOE* results were available for 22 participants.*

### Neuropsychological Testing

Mini-Mental State Examination (Folstein et al., 1975; Crum et al., 1993) and the Abbreviated Battery of Stanford–Binet IV intelligence scale (Thorndike et al., 1986), including Verbal Knowledge and Non-Verbal Fluid Reasoning subscores, were completed.

### Genetic Analyses

Saliva samples from 22 participants were assessed for *BDNF* Val66Met polymorphism and the presence of *APOE* ε4 allele, as reported previously (Jannati et al., 2017). Aliquot (700 μL) extraction of genomic DNA was performed on saliva samples collected using the Oragene Discover OGR-250 Kit (DNA Genotek Inc., Ottawa, ON, Canada). DNA was extracted from samples using standard methodology and the prepIT L2P reagent (DNA Genotek Inc., 2015). The rs6265 SNP of the *BDNF* gene, and the rs429358 and the rs7412 SNPs of the *APOE* gene were analyzed using a TaqMan single-tube genotyping assay, which uses polymerase chain reaction (PCR) amplification and a pair of fluorescent dye detectors that target the SNP. During PCR, the polymerase released the fluorescent probe into solution where it was detected using endpoint analysis in an 7900HT Real-Time instrument from Applied Biosystems, Inc. (Foster City, CA, United States).

### Transcranial Magnetic Stimulation

Two identical TMS visits (7–33 days apart; median interval = 9.5 days) were conducted. The starting times of the two visits were 0–254 min apart (interquartile range = 1–103 min; median = 12 min). The inter-visit intervals and starting-time differences for individual subjects are presented in Table 1.

All TMS procedures followed the recommended guidelines endorsed by the International Federation of Clinical Neurophysiology (Rossi et al., 2009; Rossini et al., 2015). Participants were seated in a comfortable chair with the right arm and hand in a natural pronated ∼90° angle on a pillow in front of them. They were instructed to keep their right hand as still and relaxed as possible throughout the experiment. They were also monitored for drowsiness and were asked to keep their eyes open during the TMS application. Single TMS pulses and cTBS were applied to the left primary motor cortex (M1) at 120% of individual resting motor threshold (RMT) and 80% of active motor threshold (AMT), respectively, as biphasic pulses with an antero-posterior–postero-anterior (AP-PA) induced current direction using a MagPro X100 stimulator and a MC-B70 Butterfly Coil (outer diameter: 97 mm; MagPro, MagVenture A/S, Farum, Denmark). The coil was held tangentially to the participant’s head surface, with the handle pointing occipitally and positioned at 45° relative to the mid-sagittal axis of the participant’s head. The optimal spot for the maximal responses of the right first dorsal interosseous (FDI) muscle (“motor hotspot”) was localized. A Polaris infrared-optical tracking system (Northern Digital Inc., Waterloo, ON, Canada) and a Brainsight TMS neuronavigation system (Rogue Research Inc., Montreal, QC, Canada) with a brain MRI template (for 21 participants) or the participant’s brain MRI (for the remaining 7 participants) was used to ensure consistent targeting throughout the experiment.

Surface electromyogram (EMG) was recorded from the right FDI with a PowerLab 4/25 data-acquisition device and LabChart 8 software (AD Instruments, Colorado Springs, CO, United States). Electrodes were placed over the FDI belly (negative) and the first interphalangeal joint of the second finger (positive). The ground electrode was placed over the ipsilateral ulnar styloid process. The TMS system delivered triggered pulses that synchronized the TMS and EMG systems. EMG signal was digitized at 1 kHz for 500 ms following each stimulus trigger and 100 ms pre-trigger, amplified with a range of ±10 mV (band-pass filter 0.3–1000 Hz).

Each TMS session began by localizing the motor hotspot for FDI and assessment of the RMT, defined as the lowest intensity of stimulation that elicited MEPs ≥ 50 μV in at least five of ten pulses in the relaxed right FDI. To assess pre-cTBS cortico-motor reactivity, three blocks of 30 single TMS pulses were applied to M1, with a 5–10 min inter-block interval and at a random 4–6 s inter-pulse interval. In each block, individual MEPs > 2.5 SD from the mean were excluded. Baseline MEP amplitude was calculated as the average of the peak-to-peak amplitude of MEPs in the three blocks. The AMT was then assessed as the lowest intensity that elicited MEPs ≥ 200 μV in at least five of ten pulses with the FDI slightly contracted. After a 5-min break, during which participants were instructed to maintain hand relaxation to control the effects of voluntary hand movements on cTBS responses (Iezzi et al., 2008), cTBS was applied as 200 bursts of three pulses at 50 Hz, repeated at 200-ms intervals for 40 s (for a total of 600 pulses). Cortico-motor reactivity was reassessed at 5, 10, 15, 20, 30, 40, 50, and 60 min post-cTBS (*T5*–*T60*).

### Statistical Analyses

Stata software version 13.1 (StataCorp, College Station, TX, United States) and MATLAB and Statistics and Machine Learning Toolbox R2016b (The MathWorks, Natick, MA, United States) were used for data analysis. Data from each TMS visit included: (a) RMT and AMT, expressed as percentage of maximum stimulator output; (b) baseline MEP amplitude, calculated as the average of baseline MEP amplitude in 3 blocks of 30 single TMS pulses; and (c) percent change in the average amplitude of 30 MEPs at T5–T60 relative to baseline (%Δ) for each participant.

The Shapiro–Wilk test found significant deviations in MEP values from normal distribution; thus, natural log-transformed, baseline-corrected MEP amplitude at each post-cTBS time point (ΔMEP) was averaged over all participants separately for each visit. The following measures were also calculated: absolute MEP modulation at T5–T60 (|ΔMEP|), maximum suppression and maximum modulation of MEPs during 60 min post-cTBS, area under-the-curve (AUC) and the absolute AUC value (|AUC|) of ΔMEPs over T0–T10, …, and T0–T60 intervals. Cumulative AUC and |AUC| measures up to each time-point were calculated as the summed products of the average ΔMEP and the average |ΔMEP|, respectively, across each two consecutive time-points and the time in minutes between them.

Grand-average values for all cTBS measures were calculated separately for both visits and were compared against zero using one-sample *t-*tests. Visit-B *minus* visit-A difference (Δ_B-A_) and |Δ_B-A_| were calculated for each neurophysiological measure (Table 3). All analyses were two-tailed, and the α level was set to 0.05. When explicitly noted, false discovery rate (FDR) was used to adjust *p*-values for multiple testing (Benjamini and Hochberg, 1995; Benjamini and Yekutieli, 2001).

**Table 3:** Neurophysiological measures (mean ± SD) and their test-retest reliability for the whole sample (*N* = 28).

								Reproducibility-adjusted effect sizes		
		Visit A	Visit B	Δ_B-A_	|Δ_B-A_|	ICC	*p*			
								Cohen’s *d* = 0.2	Cohen’s *d* = 0.5	Cohen’s *d* = 0.8
Motor threshold (% MSO)										
	RMT	35.3 ± 7.6	35.9 ± 7.7	0.6 ± 2.2	1.8 ± 1.4	**0**.**96**	<0.001	0.20	0.50	0.79
	AMT	25.9 ± 5.2	25.7 ± 4.6	-0.2 ± 1.8	1.5 ± 1.1	**0**.**93**	<0.001	0.20	0.49	0.78
Baseline MEP amplitude (mV)		1.3 ± 1.5	1.1 ± 1.0	-0.2 ± 1.0	0.6 ± 0.8	**0**.**70**	<0.001	0.18	0.46	0.72
Post-cTBS ΔMEP	
	T5	-0.05 ± 0.3	-0.07 ± 0.4	-0.02 ± 0.5	0.41 ± 0.3	0.16	0.213	0.13	0.31	0.48
	T10	-0.02 ± 0.4	0.08 ± 0.4	0.11 ± 0.5	0.42 ± 0.3	0.11	0.289	0.12	0.28	0.44
	T15	0.09 ± 0.3	0.00 ± 0.3	-0.09 ± 0.5	0.40 ± 0.3	-0.16	0.791	-	-	-
	T20	0.10 ± 0.3	0.03 ± 0.4	-0.07 ± 0.5	0.39 ± 0.3	0.20	0.157	0.13	0.33	0.51
	T30	0.07 ± 0.4	-0.02 ± 0.5	-0.09 ± 0.5	0.36 ± 0.4	**0**.**37**	0.024	0.16	0.39	0.61
	T40	0.07 ± 0.4	-0.06 ± 0.5	-0.14 ± 0.5	0.40 ± 0.3	0.26	0.076	0.14	0.35	0.55
	T50	0.08 ± 0.4	0.07 ± 0.5	-0.01 ± 0.5	0.37 ± 0.3	**0**.**53**	0.002	0.17	0.42	0.67
	T60	-0.04 ± 0.5	0.03 ± 0.6	0.08 ± 0.8	0.61 ± 0.5	-0.08	0.653	-	-	-
Maximum suppression		-0.50 ± 0.5	-0.53 ± 0.5	-0.03 ± 0.6	0.45 ± 0.3	**0**.**38**	0.024	0.16	0.39	0.61
Post-cTBS |ΔMEP|	
	T5	0.29 ± 0.2	0.33 ± 0.2	0.04 ± 0.3	0.29 ± 0.2	-0.27	0.912	-	-	-
	T10	0.31 ± 0.2	0.28 ± 0.2	-0.03 ± 0.4	0.27 ± 0.2	-0.18	0.815	-	-	-
	T15	0.26 ± 0.2	0.26 ± 0.2	0.00 ± 0.3	0.21 ± 0.1	0.11	0.297	0.11	0.28	0.44
	T20	0.29 ± 0.2	0.33 ± 0.3	0.03 ± 0.3	0.22 ± 0.1	0.29	0.068	0.15	0.36	0.57
	T30	0.33 ± 0.3	0.37 ± 0.3	0.03 ± 0.3	0.20 ± 0.2	**0**.**50**	0.003	0.17	0.42	0.66
	T40	0.32 ± 0.2	0.32 ± 0.3	0.00 ± 0.4	0.24 ± 0.3	0.17	0.190	0.13	0.32	0.49
	T50	0.33 ± 0.3	0.35 ± 0.3	0.02 ± 0.4	0.29 ± 0.2	**0**.**34**	0.037	0.15	0.38	0.59
	T60	0.37 ± 0.3	0.44 ± 0.4	0.07 ± 0.5	0.35 ± 0.3	-0.03	0.567	-	-	-
Maximum modulation (|ΔMEP|)		0.76 ± 0.4	0.86 ± 0.4	0.10 ± 0.4	0.35 ± 0.3	**0**.**31**	0.045	0.15	0.37	0.58
AUC of ΔMEPs	
	T0–T10	-0.33 ± 2.3	-0.15 ± 2.4	0.18 ± 3.1	2.55 ± 1.6	0.13	0.257	0.12	0.29	0.46
	T0–T15	-0.16 ± 3.6	0.05 ± 3.4	0.21 ± 4.6	3.94 ± 2.2	0.15	0.228	0.12	0.31	0.48
	T0–T20	0.31 ± 4.7	0.10 ± 4.6	-0.20 ± 6.0	5.14 ± 2.9	0.18	0.183	0.13	0.32	0.50
	T0–T30	1.13 ± 7.2	0.15 ± 8.0	-0.99 ± 9.2	7.41 ± 5.4	0.27	0.078	0.14	0.36	0.56
	T0–T40	1.85 ± 10.2	-0.26 ± 11.9	-2.12 ± 13.2	10.28 ± 8.3	0.29	0.061	0.15	0.36	0.57
	T0–T50	2.60 ± 13.3	-0.25 ± 15.4	-2.85 ± 16.2	12.70 ± 10.2	**0**.**36**	0.026	0.16	0.38	0.60
	T0–T60	2.77 ± 16.5	0.26 ± 19.1	-2.51 ± 19.7	15.94 ± 11.5	**0**.**40**	0.018	0.16	0.39	0.62
|AUC| of ΔMEP	
	T0–T10	2.22 ± 1.2	2.36 ± 1.4	0.14 ± 2.1	1.74 ± 1.2	-0.35	0.961	-	-	-
	T0–T15	3.63 ± 1.7	3.70 ± 2.1	0.07 ± 3.1	2.51 ± 1.8	-0.32	0.946	-	-	-
	T0–T20	5.02 ± 2.1	5.16 ± 2.7	0.14 ± 3.5	2.72 ± 2.2	-0.08	0.647	-	-	-
	T0–T30	8.15 ± 3.3	8.64 ± 4.1	0.48 ± 4.4	3.48 ± 2.7	0.31	0.053	0.15	0.37	0.58
	T0–T40	11.42 ± 5.0	12.05 ± 6.2	0.64 ± 6.2	4.66 ± 4.0	**0**.**39**	0.018	0.16	0.39	0.61
	T0–T50	14.66 ± 6.7	15.37 ± 8.3	0.71 ± 7.9	5.96 ± 5.2	**0**.**45**	0.008	0.16	0.41	0.64
	T0–T60	18.17 ± 8.1	19.33 ± 10.6	1.16 ± 9.9	6.00 ± 0.1	**0**.**46**	0.007	0.16	0.41	0.64


*AUC and |AUC| of ΔMEPs were calculated as the summed products of the average ΔMEP and the average |ΔMEP|, respectively, across two consecutive time-points and the time in minutes between them. The ICC values with *p* < 0.05 are highlighted in bold font. Abbreviations: Δ_*B-A*_, Visit B minus Visit A; |Δ_*B-A*_|, absolute inter-visit difference; AMT, active motor threshold; AUC, area under-the-curve; ΔMEP, natural log-transformed, baseline-corrected MEP amplitude; ICC, intraclass correlation coefficient; MEP, motor evoked potential; MSO, maximum stimulator output; RMT, resting motor threshold; T0–Tn, over the first n minutes following cTBS.*

ΔMEPs at T10 and T40 were previously found to be the best predictors of *inter*-individual variability in cTBS aftereffects in visit-A (Jannati et al., 2017). Thus, to assess the effect of potential covariates on the *intra*-individual variability of cTBS aftereffects at T10 and T40, we conducted linear mixed-effects (LME) regression analyses with Δ*MEPs at T10* or *T40* as dependent variable, *Visit* (visit-A vs. visit-B) as a within-subject factor, and potential covariates including RMT, AMT, baseline MEP amplitude, number of days between the two visits (*Inter-visit Interval*), and the absolute inter-visit difference in starting time (in minutes) (*Time Difference*) as between-subject factors. Based on previous studies that found in many situations a regression model is likely to be reliable when the number of candidate predictors is smaller than one-tenth of the number of subjects (Harrell, 2015) (p. 72), up to three between-subjects predictors were considered for simultaneous inclusion in any regression model.

To assess test–retest reliability, intraclass correlation coefficients (ICCs) (Portney and Watkins, 2009) were calculated in the form of absolute agreement between the two visits for all neurophysiological measures. ICCs were calculated using a two-way mixed-effects model, with fixed column (C) effects and random row (R) effects (McGraw and Wong, 1996):

$$\mathrm{ICC}(A,\;1)=\frac{MS_{R}-MS_{E}}{MS_{R}+(k-1)MS_{E}+\frac{k}{n}(MS_{C}-MS_{E})}$$

where ICC(A,1) represents the degree of absolute agreement of measurements made under the two fixed levels of the column factor. *k* = the number of raters/measurements per subject; *MS_R_* = mean square for rows (representing the individual subjects); *MS_E_* = mean square error; *MS_C_* = mean square for columns (representing the two visits); *n* = the number of subjects.

Using this formula, ICC = 1 indicates maximum reliability and ICC ≤ 0 indicates no reliability [in the case that the within-group variance is equal to or higher than the between-groups variance (Kenny et al., 2002)]. ICC values were interpreted as follows (Portney and Watkins, 2009): (i) ICC < 0.25: very low to no reliability; (ii) 0.25 ≤ ICC < 0.50: low reliability; (iii) 0.50 ≤ ICC < 0.75: moderate reliability; and (iv) ICC ≥ 0.75: high reliability. ICC values were statistically compared using two-way mixed-effects *F* statistics (McGraw and Wong, 1996, Table 8). The effects on the ICCs of covariates that had a significant effect on ΔMEPs were assessed by including the covariate in the corresponding mixed-effects regression model and re-calculating the residual intraclass correlation.

Lack of reliability of a measure of interest attenuates the observed effect size compared to the population parameter (Hunter and Schmidt, 1994). Following previously applied methodology (Friedman, 1968; Wright, 2014; Fried et al., 2017), we assessed how test–retest reliability (or lack thereof) of TMS measures would attenuate small, medium, and large effect sizes, i.e., Cohen’s *d* values of 0.2, 0.5, and 0.8 (Cohen, 1992), respectively, which assume perfect reproducibility. First, each *idealized* Cohen’s *d* is converted to an *r* (Cohen, 1988) (p. 23):

$$r_{\mathrm{IDEALIZED}}=\frac{d_{\mathrm{IDEALIZED}}}{\sqrt{d_{\mathrm{IDEALIZED}}^{2}+4}}$$

This idealized *r* is then *adjusted* for unreliability using the ICC (Wright, 2014):

$$r_{\mathrm{ADJUSTED}}^{2}=r_{\mathrm{IDEALIZED}}^{2}*\sqrt{ICC}$$

Finally, the adjusted *r* is converted back to an adjusted *d* (Friedman, 1968) (p. 246):

$$d_{\mathrm{ADJUSTED}}=\frac{2*r_{\mathrm{ADJUSTED}}}{\sqrt{1-r_{\mathrm{ADJUSTED}}^{2}}}$$

### Exploratory Analyses

Though unintended, the age of our participants conformed to a bimodal distribution. Thus, to explore the impact of age on our reliability measures, the total sample was subdivided into two distinct age groups with a 10-year gap and a ∼27-year difference in mean age: a *Younger* group with age < 35 (*n* = 16; range: 21–34; mean ± SD, 25.3 ± 4.3) and an *Older* group with age ≥ 45 (*n* = 12; range: 45–65; mean ± SD, 52.1 ± 6.5). To explore the effect of age on the test–retest reliability of cTBS aftereffects: (i) the ICC values of TMS measures were calculated separately for the two age groups; (ii) separate repeated-measures analyses of variance (Rm-ANOVAs) were conducted with *ΔMEP at T10* or *T40* as dependent variable, *Age Group* as a between-subjects factor, *Visit* as a within-subject factor, and *Age Group* × *Visit* interaction. Because the proportion of Hispanic participants was significantly higher in the Younger group than in the Older group, we assessed the effect of *Ethnicity* as a categorical covariate in these Rm-ANOVAs. We also re-calculated all the ICCs for the Younger group while controlling for *Ethnicity*.

To explore the roles of *BDNF* and *APOE* SNPs in the reliability of TMS measures, we calculated the ICC values of neurophysiological measures over the two visits separately for participants with *BDNF* Val/Val (Met–; *n* = 14) and Val/Met (Met+; *n* = 8) genotypes as well as for those with *APOE* ε2/ε3 or ε3/ε3 (ε4–; *n* = 12) and *APOE* ε2/ε4 or ε3/ε4 (ε4+; *n* = 10) genotypes.

Because *BDNF* was previously found to influence the cTBS aftereffect at T10 (Jannati et al., 2017), we assessed the cTBS aftereffects separately for *BDNF* Met– and Met+ participants in each visit and conducted a Rm-ANOVA with *ΔMEP at T10* as dependent variable, *BDNF Status* (Met– vs. Met+) as a between-subjects factor, *Visit* (visit-A vs. visit-B) as a within-subject factor, and *BDNF Status* × *Visit* interaction. Further, we assessed the effect of *BDNF Status* as a covariate in the LME regression analyses at T10.

## Results

Demographics, neuropsychological measures, inter-visit interval, starting times of the two visits, and inter-visit differences in starting time for individual participants are presented in Table 1. Statistical comparisons of these measures between the two age groups are presented in Table 2.

### Genetic Analyses

Available *BDNF* and *APOE* results and comparisons of all available measures between *BDNF*/*APOE* subgroups are presented in Tables 1 and 2, respectively.

Among 22 participants with available DNA results, the frequencies of *BDNF* Val/Val and Val/Met genotypes were 0.64 and 0.36, respectively, while the frequencies of *APOE* ε2/ε3, ε3/ε3, and ε3/ε4 genotypes were 0.14, 0.41, and 0.46, respectively. *BDNF* and *APOE* subgroups were comparable in all available measures (Table 2).

### Baseline Neurophysiological Measures

The RMT, AMT, and baseline MEP amplitude in each visit and their inter-visit differences are summarized in Tables 2 and 3, respectively.

There were no significant differences in any of the baseline neurophysiological measures in either visit between age or genetic subgroups (Table 2). There was also no significant difference between the two visits in any of the baseline neurophysiological measures for the whole sample (*p*’s > 0.14), for each age group (*p*’s > 0.17), or for each *BDNF* (*p*’s > 0.08) or *APOE* (*p*’s > 0.28) subgroup.

The LME regression analyses of ΔMEP at T10 found a significant, negative effect of *Time Difference* in all models (*p*’s < 0.025), but no significant effect of any of the baseline neurophysiological measures or *Visit* (*p*’s > 0.21). LME regression analyses of ΔMEP at no other time point found a significant effect of *Time Difference* (*p*’s > 0.41).

### cTBS-Induced Plasticity Results

**FIGURE 1:**
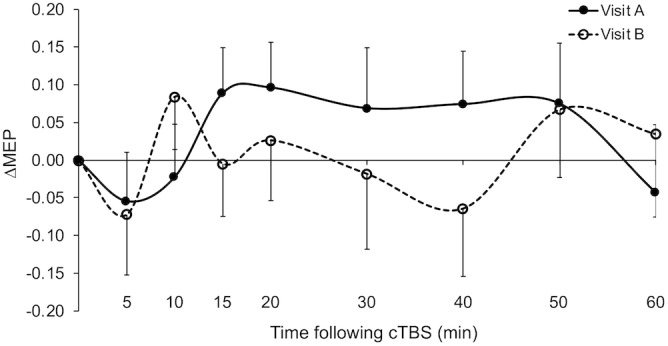
Grand-average ΔMEPs recorded from the right FDI muscle at 5 to 60 min following cTBS of the left primary motor cortex in two identical visits. The ΔMEPs did not significantly differ from zero at any post-cTBS time point in either visit (*p*’s > 0.11). Error bars represent standard error of the mean. cTBS, continuous theta-burst stimulation; ΔMEP, natural log-transformed, baseline-corrected amplitudes of the motor evoked potential; FDI, first dorsal interosseous.

Grand-average ΔMEPs in visits A and B are shown in Figure 1. ΔMEP and |ΔMEP| values, maximum suppression and maximum modulation as well as their inter-visit differences are summarized in Table 3. Grand-average ΔMEPs did not significantly differ from zero at any time point in either visit (*p*’s > 0.11). There was also no significant difference in grand-average ΔMEP or |ΔMEP| between the two visits at any time point (*p*’s > 0.16).

**FIGURE 2:**
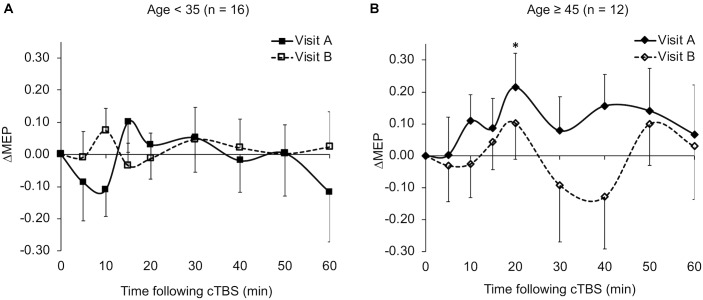
Average ΔMEPs recorded from the right FDI muscle at 5 to 60 min following cTBS of the left primary motor cortex in two identical visits in the Younger **(A)** and Older **(B)** groups. The ΔMEPs did not significantly differ from zero at any time point in either visit in the Younger group (*p*’s > 0.06). ^∗^The ΔMEPs in the Older group were significantly greater than zero at T20 in visit-A (*P*_FDR_ = 0.029), but not at any other time point in either visit (*p*’s > 0.18). Error bars represent standard error of the mean. cTBS, continuous theta-burst stimulation; ΔMEP, natural log-transformed, baseline-corrected amplitudes of the motor evoked potential; FDI, first dorsal interosseous; FDR, false discovery rate.

The ΔMEPs in the Older group were significantly greater than zero at T20 in visit-A (*P*_FDR_ = 0.029), but not at any other time point in either visit (*p*’s > 0.18). The ΔMEPs in the Younger group did not significantly differ from zero at any time point in either visit (*p*’s > 0.06). The ΔMEPs in the *BDNF* Met– group were significantly less than zero at T10 in visit-A (*P*_FDR_ = 0.042), but not at any other time point in either visit (*p*’s > 0.14). The ΔMEPs in the *BDNF* Met+ group were not significantly different from zero at any time point in either visit (*P*_FDR_’s > 0.05). cTBS aftereffects in both visits for the two age subgroups and the two *BDNF* subgroups are presented in Figure 2 and 4 respectively.

The Rm-ANOVA on the ΔMEP at T10 found a significant effect of *BDNF* status, *F*(1, 20) = 8.28, *p* = 0.009, $\eta_{p}^{2}$ = 0.29, but no significant effects of Visit or *BDNF* × *Visit* interaction (*p*’s > 0.10). *BDNF* Met-carrier status had a significant positive effect in all LME regression analyses of ΔMEP at T10 (${\hat{B}}^{\prime }$s > 0.28, *p*’s < 0.027). There was no other significant effect in any of the LME models (*p*’s > 0.08).

To control for potential effects of gender, race/ethnicity, and handedness on cTBS-induced plasticity measures, we calculated ΔMEP’s at T5–T60 in a subgroup of White, non-Hispanic, and right-handed males (*n* = 11, Table 1). In this smaller, but more-homogenous subsample, ΔMEPs did not significantly differ from zero at any time point in either visit (*p*’s > 0.23).

### Test–Retest Reliability of TMS Measures

Measures of inter-visit variability and test–retest reliability for RMT, AMT, baseline MEP amplitude and cTBS measures, as well as reliability-adjusted effect sizes for each TMS measure in the whole group are presented in Table 3.

**FIGURE 3:**
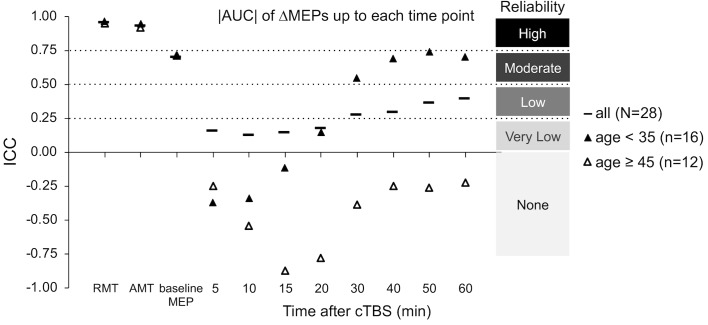
Test–retest reliability of baseline neurophysiological measures and post-cTBS ΔMEP measures separately in the Younger (age < 35, *n* = 16) and Older (age ≥ 45, *n* = 12) groups. |AUC| of ΔMEPs were calculated as the summed products of the average |ΔMEP| across each two consecutive time-points and the time in minutes between them over T0–T10, T0–15, …, T0–T60 intervals (marked by their end time point on the abscissa). The ICCs of the RMT, AMT, and baseline MEP amplitude were not significantly different between the two age groups (*p*’s > 0.19). The |AUC| of ΔMEPs were significantly more reliable in the Younger group than in the Older group over T0–T15 and beyond (*P*_FDR_’s < 0.001). The reliability of other cumulative ΔMEP measures was not significantly different between the two age groups (*p*’s > 0.058). AUC, area under-the-curve; cTBS, continuous theta-burst stimulation; FDR, false discovery rate; ΔMEP, natural log-transformed, baseline-corrected MEP amplitude; ICC, intraclass correlation coefficient; MEP, motor evoked potential; T0–T*n*, over the first *n* minutes post-cTBS.

The ICCs of baseline neurophysiological measures was not significantly different between the two age groups (*p*’s > 0.19; Figure 3). In contrast, ΔMEPs in the Younger group were significantly more reliable than in the Older group at T10 and T60 (*P*_FDR_’s < 0.015), but not at other individual time points (*P*_FDR_’s *>* 0.058). Similarly, |AUC| measures were significantly more reliable in the Younger group than in the Older group over T0–T20 and beyond (*P*_FDR_’s < 0.001; Figure 3). The reliability of other cumulative ΔMEP measures was not significantly different between the two age groups (*p*’s > 0.058). After adjusting for *Ethnicity*, none of the ICCs in the Younger group crossed our pre-defined boundaries for interpretation of ICC values (see “Materials and Methods” section).

To control for potential effects of gender, race/ethnicity, and handedness on the test–retest reliability of cTBS aftereffects, we calculated the ICC values of baseline neurophysiological measures and ΔMEP’s at T5–T60 among White, non-Hispanic, and right-handed males (*n* = 11, Table 1). The ICC values of RMT, AMT, and baseline MEP amplitude in this subgroup were 0.86 (*p* < 0.001), 0.96 (*p* < 0.001), and 0.75 (*p* = 0.003), respectively. The ICC values of ΔMEP at T5, T10, T15, T20, T30, T40, T50, and T60 in this subgroup were 0.54 (*p* = 0.031), –0.09 (*p* = 0.602), 0.18 (*p* = 0.297), 0.71 (*p* = 0.005), 0.86 (*p* < 0.001), 0.67 (*p* = 0.008), 0.79 (*p* = 0.001), and 0.17 (*p* = 0.309), respectively.

The ICCs of baseline neurophysiological measures were not significantly different between either the *BDNF* or the *APOE* subgroups (*p*’s > 0.16; Figure 5). In contrast, ΔMEPs were significantly more reliable in *BDNF* Met– participants than in *BDNF* Met+ participants at T20–T40 (*P*_FDR_’s < 0.023), but not at other time points (*p*’s > 0.24). Maximum suppression, maximum modulation, and AUC of ΔMEPs over T0–T30 and beyond were significantly more reliable in *BDNF* Met– participants than in *BDNF* Met+ participants (*P*_FDR_’s < 0.032; Figure 5). The reliability of other cumulative ΔMEP measures was not significantly different between the two *BDNF* subgroups (*P*_FDR_ > 0.21).

ΔMEPs were significantly more reliable in *APOE* ε4– participants than in *APOE* ε4+ participants at T5 and T20–T40 (*P*_FDR_’s < 0.024), but not at other time points (*P*_FDR_’s > 0.07). All AUC measures were significantly more reliable in *APOE* ε4– participants than in *APOE* ε4+ participants (*P*_FDR_’s < 0.021; Figure 5). There were no significant differences in the reliability of maximum suppression or maximum modulation between the two *APOE* subgroups (*P*_FDR_’s > 0.27).

## Discussion

Test–retest reliability of TMS measures influences their utility as potential neurophysiologic biomarkers or targets for therapeutic intervention. As the use of plasticity-inducing rTMS protocols becomes more common, it is necessary to investigate the magnitude and sources of their inter- and intra-individual variability. While some of the factors that contribute to the inter-individual variability of these types of plasticity metrics among healthy individuals have been identified (Cheeran et al., 2008, 2009; Antal et al., 2010; Hamada et al., 2013; Goldsworthy et al., 2014; López-Alonso et al., 2014; Nettekoven et al., 2014, 2015; Vallence et al., 2015; Suppa et al., 2016; Hordacre et al., 2017; Jannati et al., 2017), few studies have assessed the intra-individual reliability of cTBS responses (Vernet et al., 2014; Vallence et al., 2015) and no study, to our knowledge, has systematically assessed the test–retest reliability of cTBS aftereffects during 60 min post-cTBS. The present study was designed to fill this gap by assessing the test–retest reliability of cTBS aftereffects at 5- or 10-minute intervals (T5–T60) and of cumulative cTBS aftereffects during 60 min post-cTBS in healthy adults. Furthermore, in order to provide guidance and reference for future studies, we calculated adjusted effect sizes that take into account the test–retest reliability of cTBS measures. Finally, we explored the influences of age group and common SNPs in *BDNF* and *APOE* genes on the reliability of cTBS aftereffects.

### Overall Reliability of Baseline Neurophysiological Measures

Resting motor threshold had high test–retest reliability (Table 3), which was comparable with the ICC values reported in most previous studies (Carroll et al., 2001; Kimiskidis et al., 2004; Livingston and Ingersoll, 2008; Bastani and Jaberzadeh, 2012; Ngomo et al., 2012; Hinder et al., 2014; Schambra et al., 2015; Fried et al., 2017; Davila-Pérez et al., 2018) and somewhat higher than other studies that found RMT ICCs in the 0.75–0.80 range (Fleming et al., 2012; Liu and Au-Yeung, 2014; Sankarasubramanian et al., 2015; Hermsen et al., 2016). The AMT also had high test–retest reliability (Table 3), which was comparable with the results of previous studies (Ngomo et al., 2012; Hinder et al., 2014; Fried et al., 2017).

Baseline MEP amplitude had moderate test–retest reliability (Table 3). The ICC of baseline MEP amplitude found in the present study (ICC = 0.70) was moderate compared to the wide range of ICC values for baseline MEP amplitude (–0.16 to 0.87) reported in previous studies (Kamen, 2004; McDonnell et al., 2004; Christie et al., 2007; Fleming et al., 2012; Ngomo et al., 2012; Hinder et al., 2014; Hermsen et al., 2016; Fried et al., 2017; Davila-Pérez et al., 2018). Variability of baseline MEP amplitude was previously found to be associated with variability of TBS aftereffects (Hordacre et al., 2017; Fried et al., 2017). The moderate reliability of baseline MEP amplitude in the present study suggests that such variability was not the main cause of the low reliability of some of the cTBS measures reported here.

### Overall Reliability of cTBS Aftereffects

The finding that differences between the start times of the two visits influenced the cTBS aftereffects at T10 could be due to the effect of circadian rhythm on the neuromodulatory effects of rTMS arising from changes in cortical excitability and synaptic efficiency during the day (Cohen et al., 2005). While the present results cannot definitively conclude that circadian factors influenced the intra-individual variability in plasticity at T10, future studies could attempt to control for the time of day or, perhaps even better, to individualize visits to coincide with the same relative point in each subject’s circadian cycle.

The finding that T5 had one of the lowest between-visit variabilities among post-cTBS time points is consistent with the findings of a previous study (Vernet et al., 2014). Importantly, however, the low between-visit variability of cTBS aftereffects at T5 and T50 at the group level in the present study did not translate to high test–retest reliability measures, which take into account both within-individual and between-individuals variability; while T50 was the most reliable post-cTBS time point (ICC = 0.53), T5 had very low reliability (ICC = 0.16). This pattern of results underlines the importance of calculating the ICCs of TMS measures rather than relying only on measures of inter-visit variability at the group level. Further, the low ICCs at T5 and T10 indicate that the time points expected to show maximal effects of cTBS (Wischnewski and Schutter, 2015) do not necessarily exhibit high test–retest reliability. This remained true for the cumulative measures of cTBS aftereffects over the first 20 min post-cTBS.

**FIGURE 4:**
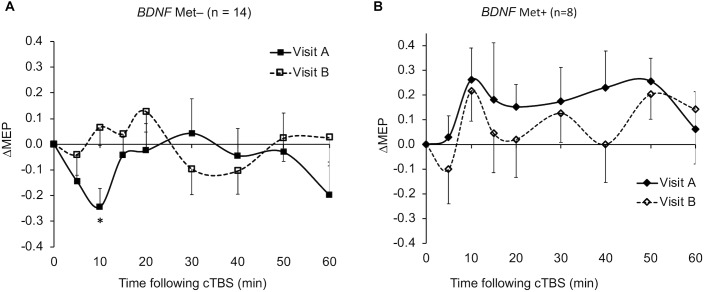
Average ΔMEPs recorded from the right FDI muscle at 5 to 60 min following cTBS of the left primary motor cortex in two identical visits in the *BDNF* Met– **(A)** and Met+ **(B)** groups. ^∗^The ΔMEPs in the *BDNF* Met– group were significantly less than zero at T10 in visit-A (*P*_FDR_ = 0.042), but not at any other time point in either visit (*p*’s > 0.14). The ΔMEPs in the *BDNF* Met+ group were not significantly different from zero at any time point in either visit (*P*_FDR_’s > 0.05). Error bars represent standard error of the mean. *BDNF*, brain-derived neurotrophic factor; *BDNF* Met–, Val66Val; *BDNF* Met+, Val66Met; cTBS, continuous theta-burst stimulation; FDR, false discovery rate; ΔMEP, natural log-transformed, baseline-corrected; MEP amplitude; MEP, motor evoked potential; Met, metionine; Val, valine.

**FIGURE 5:**
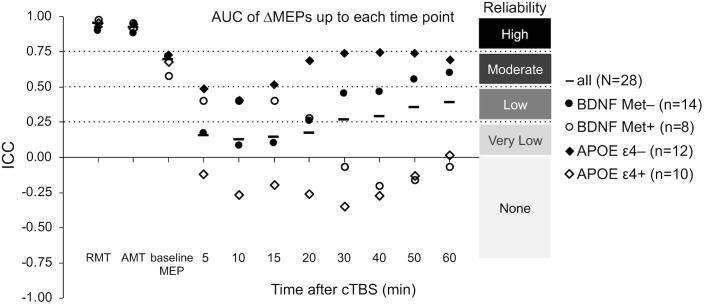
Test–retest reliability of baseline neurophysiological measures and post-cTBS ΔMEP measures separately in the *BDNF* Met–/Met+ and *APOE* ε4–/ε4+ groups. AUC of ΔMEPs were calculated as the summed products of the average ΔMEP across each two consecutive time-points and the time in minutes between them over T0–T10, T0–15, …, T0–T60 intervals (marked by their end time point on the abscissa). The ICC values of RMT, AMT, and baseline MEP amplitude were not significantly different between the two *BDNF* groups (*p*’s > 0.19) or the two *APOE* groups (*p*’s > 0.16). Maximum suppression, maximum modulation, and the AUC of ΔMEPs over T0–T30 and beyond were significantly more reliable in the *BDNF* Met– group than in the *BDNF* Met+ group (*P*_FDR_’s < 0.032). The reliability of other cumulative ΔMEP measures was not significantly different between the two *BDNF* groups (*P*_FDR_ > 0.208). All the AUC measures were significantly more reliable in the *APOE* ε4– group than in the *APOE* ε4+ group (*P*_FDR_’s < 0.021). The maximum suppression, maximum modulation were not significantly different between the two *APOE* groups (*P*_FDR_’s > 0.27). AMT, active motor threshold; *APOE*, apolipoprotein E; *APOE* ε4+, ε2/ε4 or ε3/ε4 genotype; *APOE* ε4–, ε2/ε3 or ε3/ε3 genotype; AUC, area under-the-curve; *BDNF*, brain-derived neurotrophic factor; *BDNF* Met–, Val66Val; *BDNF* Met+, Val66Met; cTBS, continuous theta-burst stimulation; FDR, false discovery rate; ICC, intraclass correlation coefficient; ΔMEP, natural log-transformed, baseline-corrected MEP amplitude; MEP, motor evoked potential; Met, metionine; RMT, resting motor threshold; T0–T*n*, over the first *n* minutes following cTBS; Val, valine.

The very low test–retest reliability of ΔMEPs at T10 (ICC = 0.11) could be due to two factors: (1) The ΔMEPs at T10 in both visits could be the most influenced by *BDNF* polymorphism (Jannati et al., 2017). Consistent facilitation of MEPs, at least numerically, in *BDNF* Met+ participants in both visits may have resulted in higher test–retest reliability of T10 ΔMEP in that subgroup (Figure 5). (2) Despite the relatively long inter-visit interval in the present study, T10 seemed to exhibit a *metaplastic*-like effect similar to those reported with shorter intervals (Maeda et al., 2000; Gentner et al., 2008; Valero-Cabré et al., 2008; Oberman et al., 2016) in the overall results (Figure 1), as well in the Younger group (Figure 2A) and the *BDNF* Met– subgroup (Figure 4A). In all three cases, the direction of the neuromodulatory effect of cTBS at T10 was reversed, at least numerically, from inhibitory in visit-A to facilitatory in visit-B. Such reversals, when predominant at the individual level, would substantially reduce the test–retest reliability of cTBS aftereffects at T10. Although a previous iTBS study found only inter-visit intervals shorter than 7 days to be associated with metaplastic changes after iTBS in aging adults (Fried et al., 2017), the initial cTBS in the present study may have set into motion subtle changes that were still present when the second cTBS was applied. Further, it is possible that demographic, genetic, and state-dependent factors modulate the metaplastic(-like) effects of successive TBS sessions (Opie et al., 2017).

The low test–retest reliability of several cTBS aftereffects resulted in adjustment of large- and medium effect sizes to medium and small effect sizes, respectively (Table 3). While calculating the cumulative cTBS measures improved the overall ICCs over T0–T30 and beyond (Figure 3, 5), the ICCs of the cumulative measures for the whole sample remained below 0.5. Attenuation of idealized effect sizes by this level of reproducibility indicates that detecting significant differences in cTBS responses between healthy and clinical populations may require sample sizes that are substantially larger than those used in most previous cTBS studies (Wischnewski and Schutter, 2015; Chung et al., 2016; Suppa et al., 2016), unless steps can be implemented to improve the reliability of this technique.

The finding that within White, non-Hispanic, and right-handed males (*n* = 11), none of the ΔMEPs at any time point in either visit differed significantly from zero suggests that controlling for demographic factors such as gender, race/ethnicity, and handedness is not enough to overcome the large inter-individual variability in cTBS responses in either visit. We found, despite comparable reliability of baseline neurophysiological measures, T5 and T20–T50 ΔMEPs were substantially more reliable in this more-homogenous subgroup, suggesting that heterogeneity of these demographic factors influence the reliability of cTBS aftereffects in the whole sample. While the small sample precludes definitive conclusions about the effects of gender, handedness, and race/ethnicity, these results nonetheless appear to suggest that demographic variation plays a role in the test–retest reliability of cTBS responses.

### Age and Reliability of cTBS Aftereffects

The significant *Age Group* × *Visit* interaction effect on T10 ΔMEP indicates that cTBS aftereffects at T10 in the Younger group, but not in the Older group, switched from inhibitory in visit-A to facilitatory in visit-B (Figure 2). Such reversal could be due to metaplasticity or some other state-dependent factor. The distinct patterns of cTBS aftereffects at T10 among Younger and Older groups could be due to two factors: (i) Based on animal studies that have found an age-related reduction in the efficiency of gamma-aminobutyric acid- (GABA-) mediated inhibition (Milbrandt et al., 1994; Billard et al., 1995; McQuail et al., 2012), it is possible that older participants have less-efficient GABAergic synaptic transmission, which is presumed to be involved in cTBS-induced plasticity (Stagg et al., 2009; Trippe et al., 2009). The resulting reduced inhibitory effects of cTBS, potentially combined with cumulative facilitatory effects of successive single TMS pulses (Pellicciari et al., 2016), could have resulted in facilitation of MEPs in the Older group, at least in visit-A. (ii) The finding that the Younger group, but not the Older group, showed metaplastic-like changes at T10 could be due to age-related differences in the priming effect of TBS (Opie et al., 2017), i.e., a stronger priming effect of cTBS in visit-A among younger participants.

Similarly, the finding that despite comparable ICCs of baseline neurophysiological measures in the two age groups, cTBS aftereffects were substantially less reliable in older participants (Figure 3) could be due to the age-related decrease in the efficiency of GABAergic synaptic transmission reported in animal studies (Milbrandt et al., 1994; Billard et al., 1995; McQuail et al., 2012). These results indicate that in order to retain adequate power to detect differences in cTBS measures of plasticity in future studies, it may be necessary to adjust effect sizes separately for younger and older age groups.

### *BDNF* and *APOE* Polymorphisms and Reliability of cTBS Aftereffects

Despite comparable ICC values of RMT, AMT, and baseline MEP amplitude in *BDNF* Met– and Met+ groups, cTBS aftereffects at several time points and most cumulative measures of cTBS aftereffects were substantially more reliable in *BDNF* Met– participants than in Met+ participants (Figure 5). This pattern of results could be due to the following: *BDNF* Met carrier status is known to be associated with impaired *N*-Methyl-D-aspartic acid- (NMDA-)dependent LTD (Woo et al., 2005), aberrant GABAergic synaptic transmission (Abidin et al., 2008), reduced cTBS-induced inhibition of MEPs (Chung et al., 2016), and “paradoxical” cTBS-induced facilitation of MEPs in visit-A reported in our previous study (Jannati et al., 2017) and a few other studies (Gentner et al., 2008; Goldsworthy et al., 2012; Hellriegel et al., 2012; Brownjohn et al., 2014). The finding that *BDNF* Met+ participants showed MEP facilitation, at least numerically, at T10 in both visits (Figure 4B) supports an association between *BDNF* Met+ status and facilitatory response to cTBS at T10 in the present sample. The noticeably lower test–retest reliability of cTBS aftereffects in *BDNF* Met+ participants (Figure 5) could be due to the less-efficient cTBS-induced plasticity caused by aberrant GABAergic inhibition (Abidin et al., 2008), assumed to be involved in the LTD-like effects of cTBS (Stagg et al., 2009; Trippe et al., 2009).

Despite comparable ICC values of RMT, AMT, and baseline MEP amplitude in *APOE* ε4– and ε4+ participants, cTBS aftereffects at several time points and most cumulative measures of cTBS aftereffects were substantially more reliable in *APOE* ε4– participants than in *APOE* ε4+ participants (Figure 5). These results could be due to the influence of *APOE* ε4 on NMDA-mediated synaptic plasticity, which has been found to be involved in TBS aftereffects (Huang et al., 2007; Chen et al., 2010). These results are also consistent with the less-efficient rTMS-induced activation of brain networks in *APOE* ε4 carriers (Peña-Gomez et al., 2012).

The small number of participants in the *BDNF* and *APOE* subgroups study limits the generalizability of the present findings on genetic influences on the test–retest reliability of cTBS measures. Assuming that the noticeable differences in reliability of cTBS aftereffects in *BDNF* and *APOE* subgroups observed here (Figure 5) are confirmed in future studies, it would be advantageous to consider the expected proportions of *BDNF* and *APOE* subgroups and adjust effect sizes for each SNP subgroup accordingly. For example, the minor allele frequencies of rs6265 (*BDNF*), rs429358 (*APOE*), and rs7412 (*APOE*) SNPs in the admixed American population in the 1000 Genomes Project (Auton et al., 2015) are 0.1527, 0.1037, and 0.0476, respectively. As long as that the SNP frequencies among participants do not significantly deviate from Hardy–Weinberg equilibrium proportions (Guo and Thompson, 1992; Wigginton et al., 2005), these frequencies provide good approximations to the frequencies of minor *BDNF* and *APOE* alleles in future cTBS studies.

In comparisons of test–retest reliability of cTBS measures between age or genetic subgroups, the finding that the subgroups in each case were comparable in gender, race/ethnicity, handedness, and the reliability of baseline neurophysiological measures indicates that differences in heterogeneity of demographic factors and baseline cortical excitability did not play a major role in the observed differences in test–retest reliability of cTBS aftereffects.

In addition to considering age, genetic polymorphisms, inter-visit interval (Fried et al., 2017), the time of day (Cohen et al., 2005), and the use of neuronavigation (Julkunen et al., 2009), other factors that could improve the test–retest reliability of TMS measures include: ensuring comparable blood glucose levels and caffeine intake before and during each visit (Specterman et al., 2005; Cerqueira et al., 2006; Badawy et al., 2013), comparable amount and quality of sleep the night before each visit (Civardi et al., 2001; Kreuzer et al., 2011), comparable intensity and duration of exercise before each visit (Samii et al., 1997; Lentz and Nielsen, 2002), comparable phase of the menstrual cycle across visits (Smith et al., 1999; Hattemer et al., 2007), the use of robotic arms such as the TMS-Robot (Axilum Robotics, Schiltigheim, France), which can reduce trial-to-trial variability of MEP amplitude (Foucher et al., 2012), comparable baseline MEP amplitude across plasticity visits (Fried et al., 2017), and implementing closed-loop systems that trigger TMS pulses timed to real-time, EEG-defined indices of brain states (Zrenner et al., 2016, 2018).

## Conclusion

The present study assessed the test–retest reliability of cTBS aftereffects in healthy adults. cTBS aftereffects at most individual time points had low to moderate reliability. Cumulative cTBS measures over the first 30 min and beyond were relatively more reliable. Effect sizes adjusted for reliability of cTBS aftereffects are provided to help future studies retain adequate power for comparing M1 cTBS responses between healthy and clinical populations. Those calculations resulted in adjustment of several large and medium effect sizes to medium and small effect sizes, respectively, thereby substantially increasing the estimates of the required sample size to detect a significant difference in cTBS responses between healthy and clinical populations. Exploratory analyses found cTBS aftereffects were substantially more reliable in younger participants (age < 35 years) and those with *BDNF* Met– and *APOE* ε4– genotypes.

## Ethics Statement

This study was approved by the Institutional Review Board at Beth Israel Deaconess Medical Center in accordance with the Declaration of Helsinki. All participants provided written informed consent prior to enrollment and received monetary compensation upon completion.

## Author Contributions

AJ, AR, and AP-L conceived and designed the study. AJ and GB collected the data. AJ analyzed the data and drafted the manuscript. AJ, PF, AR, and AP-L interpreted the data. All authors revised the manuscript, approved the final version, and agreed to be accountable for the content of the work.

## Conflict of Interest Statement

AP-L serves on the scientific advisory boards for Neuronix, Starlab Neuroscience, Neuroelectrics, Constant Therapy, Cognito, NovaVision, and Neosync, and is listed as an inventor on several issued and pending patents on real-time integration of TMS with EEG and MRI. AR is a founder and advisor for Neuromotion, serves on the medical advisory board for NeuroRex, and is listed as an inventor on a patent related to integration of TMS and EEG. The remaining authors declare that the research was conducted in the absence of any commercial or financial relationships that could be construed as a potential conflict of interest.

## Abbreviations

%Δ: percent change from the baseline
Δ_B-A_: visit-B *minus* visit-A
ΔMEP: natural log-transformed, baseline-corrected amplitude of motor evoked potentials
*APOE*: apolipoprotein E
AUC: area under-the-curve
*BDNF*: brain-derived neurotrophic factor
cTBS: continuous theta-burst stimulation
FDR: false discovery rate
GABA: gamma-aminobutyric acid
ICC: intraclass correlation coefficient
iTBS: intermittent theta-burst stimulation
LME: linear mixed-effects regression analysis
LTD: long-term depression
LTP: long-term potentiation
NMDA: *N*-Methyl-D-aspartic acid
PCR: polymerase chain reaction
Rm-ANOVA: repeated-measures analysis of variance
SNP: single-nucleotide polymorphism
T0–T*n*: over the first *n* minutes following cTBS
TMS: transcranial magnetic stimulation
T*n*: at *n* minutes post-cTBS
